# siRNAs Targeting Growth Factor Receptor and Anti-Apoptotic Genes Synergistically Kill Breast Cancer Cells through Inhibition of MAPK and PI-3 Kinase Pathways

**DOI:** 10.3390/biomedicines6030073

**Published:** 2018-06-22

**Authors:** Nur Izyani Kamaruzman, Snigdha Tiash, Maeirah Ashaie, Ezharul Hoque Chowdhury

**Affiliations:** Jeffrey Cheah School of Medicine and Health Sciences, Faculty of Medicine, Nursing and Health Sciences, Monash University, Wellington Rd & Blackburn Rd, Clayton, VIC 3800, Australia; nurizyanikamaruzman@gmail.com (N.I.K.); snigdha.tiash@unimelb.edu.au (S.T.); maira.ashaie@gmail.com (M.A.)

**Keywords:** carbonate apatite nanoparticle, siRNA, estrogen receptor (ER), mitogen-activated protein kinase (MAPK), protein kinase B (AKT), breast cancer

## Abstract

Breast cancer, the second leading cause of female deaths worldwide, is usually treated with cytotoxic drugs, accompanied by adverse side-effects, development of chemoresistance and relapse of disease condition. Survival and proliferation of the cancer cells are greatly empowered by over-expression or over-activation of growth factor receptors and anti-apoptotic factors. Identification of these key players that cross-talk to each other, and subsequently, knockdown with their respective siRNAs in a synchronous manner could be a promising approach to precisely treat the cancer. Since siRNAs demonstrate limited cell permeability and unfavorable pharmacokinetic behaviors, pH-sensitive nanoparticles of carbonate apatite were employed to efficiently carry the siRNAs in vitro and in vivo. By delivering selective siRNAs against the mRNA transcripts of the growth factor receptors, such as ER, ERBB2 (HER2), EGFR and IGFR, and anti-apoptotic protein, such as BCL2 in human (MCF-7 and MDA-MB-231) and murine (4T1) breast cancer cell lines, we found that ESR1 along with BCL-2, or with ERBB2 and EGFR critically contributes to the growth/survival of the cancer cells by activating the MAPK and PI-3 kinase pathways. Furthermore, intravenous delivery of the selected siRNAs aiming to suppress the expression of ER/BCL2 and ER/ERBB2/EGFR groups of proteins led to a significant retardation in tumor growth in a 4T1-induced syngeneic mouse model.

## 1. Introduction

Breast cancer is a life-threatening cancer with estimated 1.67 million new cases of the cancer diagnosed in 2012, worldwide [1]. Breast cancer cells may metastasize from the ducts or lobules to other body parts by invading the blood and lymphatic vessels; therefore, sophisticated treatments are needed to destroy them. Adverse toxic effects of chemotherapeutic drugs that lead to their dose limitation on breast cancer patients finally reduce their effectiveness. Some other cases showed chemoresistance and consequential relapse of the disease [2]. A challenging issue is to design drugs that are selectively cytotoxic to neoplastic cells without exerting harmful effects on healthy cells.

Conversion of proto-oncogenes into oncogenes via mutations is one of the prominent causes of the disease, promoting overexpression of growth factor receptors and subsequent cross-talks among their downstream signaling cascades, and thus leading to proliferation and survival of cancer cells [3]. Estrogen receptor (ER), a hormone-dependent receptor that requires estrogen to bind to it and transmits intracellular signals plays various critical roles in breast cancer proliferation and survival. Among American women with breast cancer, 77% of the cases are found to be positive for the ER expression [4]. Although expression of the classical ER was seen on the nuclear membrane of cells, studies on MCF-7 breast cancer cells have suggested some pools of ER to be available at the plasma membrane and cytoplasmic region [5]. Intriguingly, the collaboration between the signaling pathways of ER and other growth factor receptors (e.g., human epidermal growth factor receptor 2, HER2; epidermal growth factor receptor, EGFR; and insulin-like growth factor receptor, IGFR) have been reported to render the hormonal therapy resistant in breast cancer [6]. ER may also function as the transcription regulator of other genes responsible for cell growth in normal and cancerous states [7]. Escalated growth signals cross-talk with death signals to inhibit apoptosis as well [8]. Thus, silencing the expression of ER and other growth factor receptor genes concurrently might be a promising approach to breast cancer treatment.

Nanotechnology is currently being explored in the development of nano-size drugs to efficiently deliver chemotherapy drugs to breast cancer cells and to address the toxicity concern in relation to administration of higher doses of the drugs [9]. The nanoformulation of an anti-cancer drug is also advantageous to overcome the chemoresistance of breast cancer cells, probably by enhancing passive targeting of the drug [10]. An interesting example of nanomedicine involves monoclonal antibody (mAb) as a drug carrier, targeting a specific antigen or receptor expressed on the cancer cells [11,12]. mAb can also be designed as a therapeutic drug, such as Herceptin (trastuzumab), a recombinant humanized mAb that binds HER2 and thus inhibits cancer cell growth [13]. In addition, gene therapy through nucleic acid-based therapeutics such as the introduction of exogenous small interfering RNA (siRNA) into the breast cancer cells might be harnessed in order to overcome dose limitation of the chemotherapeutic drugs in clinical settings. 

siRNA is a duplex RNA of ~21–28 nucleotides that selectively degrades a mRNA transcript and thereby blocks its translation into a particular protein. Once in cytoplasm, siRNA is incorporated into a multiprotein RNA-induced silencing complex (RISC) and unwound into a single-stranded (antisense) RNA that finally guides and selectively degrades the complementary mRNA with the help of Argonaute-2 of RISC [14]. Despite the remarkable potency of siRNA in silencing specific gene expression, its half-life is too short as a result of degradation by blood nucleases, with even limited permeability across the target cell membrane predominantly owing to its negatively charged phosphate backbone that repels the anionic membrane [14]. Therefore, a suitable nano-carrier to transport siRNA molecules into tumor cells via endocytosis and subsequently release them in cytoplasm is the prerequisite for achieving the maximum therapeutic outcomes from siRNA-mediated cleavage of selective mRNA(s).

We developed pH-sensitive inorganic nanoparticles of carbonate apatite (CA) as smart carriers of siRNAs and plasmid DNAs in vitro as well as in vivo. Strong electrostatic binding affinity towards nucleic acids under physiological pH, but fast dissolution rate at endosomal acidic pH made these nanoparticles highly efficient to carry the loaded siRNAs or plasmids into cytosol, facilitating the subsequent action of mRNA knockdown or nuclear translocation, respectively [15,16,17,18,19]. The role of *ROS1* gene in growth/survival and chemo-sensitization of breast cancer cells (MCF-7 and 4T1) was validated through intracellular delivery of ROS1 siRNA after being embedded into these nanoparticles [19]. Furthermore, intravenous delivery of siRNA targeting *ROS1* gene using the nanoparticles led to a reduction in tumor volume, with a synergistic effect following co-delivery with an anti-cancer drug (doxorubicin) in a syngeneic mouse model [20]. In order to identify the major cross-talks among growth factor receptors, ER, ERBB2, IGFR and EGFR, and anti-apoptotic protein, BCL2 in promoting growth/survival of different breast cancer cell lines, we delivered the siRNAs targeting those endogenous proteins individually as well as in combinations with help of the nanoparticles into MCF-7, MDA-MB-231 and 4T1 cells, and found that ER along with either BCL2, or ERBB2 and EGFR critically contributes to the growth/survival of the cancer cells by activating the mitogen-activated protein kinase (MAPK) and phophoinositide 3-kinase (PI3K)/protein kinase B (AKT) pathways. Furthermore, systemic delivery of the nanoparticles carrying the siRNAs to suppress the expression of ER/BCL-2 and ER/ERBB2/EGFR groups of proteins resulted in a notable and sustainable decrease in tumor growth in a 4T1-induced syngeneic mouse model.

## 2. Materials and Methods

### 2.1. Reagents

Dulbecco’s modified Eagle medium (DMEM), DMEM powder, foetal bovine serum (FBS), TrypLE Express enzyme (1×) (trypsin-EDTA) and penicillin/streptomycin were obtained from Gibco BRL (Carlsbad, CA, USA). Calcium chloride dehydrate (CaCl_2_·2H_2_O), sodium bicarbonate, hepes, dimethyl sulphoxide (DMSO) and thiazolyl blue tetrazolium bromide (MTT) were from Sigma-Aldrich (St. Louis, MO, USA). Western blots were carried out by the antibodies purchased from Cell Signaling Technology^®^ (Danvers, MA): Estrogen Receptor α (D8H8) Rabbit mAb, Phospho-Estrogen Receptor α (Ser167) (D1A3) Rabbit mAb, p44/42 MAPK (Erk1/2) (137F5) Rabbit mAb, Phospho-p44/42 MAPK (Erk1/2) (Thr202/Tyr204) (D13.14.4E) XP^®^ Rabbit mAb, Akt (pan) (C67E7) Rabbit mAb, Phospho-Akt (Ser473) and GAPDH (14C10) Rabbit mAb.

### 2.2. siRNA Sequence

The validated anti-ER (ESR1), anti-ERBB2 (HER-2), anti-IGFR (IGF1R), anti-EGFR and anti-BCL2 siRNAs were purchased from QIAGEN (Valencia, CA, USA) with target sequence of 5′-GAGACTTGAATTAATAAGTGA-3′, 5′-AACAAAGAAATCTTAGACGAA-3′, 5′-ATGGAGAATAATCCAGTCCTA-3′, 5′-TACGAATATTAAACACTTCAA-3′, and 5′-AACCGGGAGATAGTGATG-3′, respectively. The negative control siRNA was also bought from QIAGEN. The 1 nmol siRNAs were supplied in lyophilized form and were reconstituted according to manufacturer’s instruction to make 10 μM stock and stored at −20 °C.

### 2.3. Cell Culture and Seeding

MCF7, MDA-MB-231 and 4T1 cell lines were cultured on 75 cm^3^ tissue culture flasks in Dulbecco’s modified Eagle’s medium supplemented with 10% fetal bovine serum (FBS), 50 μg/mL penicillin and 50 μg/mL streptomycin and 10% Hepes at 37 °C in a humidified 5% CO_2_-containing atmosphere. Cells were trypsinised at an exponential growth rate and fresh medium was added. Cells were centrifuged at 1000 rpm for 5 min and supernatant was discarded. Cells pellet was resuspended in fresh medium and haemocytometer was used to perform cell counting. 50,000 cells were seeded into each well of the 24-well plate (Nunc, Roskilde, Denmark). Cells were allowed overnight for attachment and growth at 37 °C in a humidified 5% CO_2_-containing atmosphere.

### 2.4. Imaging of Particles with Scanning Elentron Microscope (SEM)

CA nanoparticles were prepared as mentioned above, with the incorporation of appropriate amounts of CaCl_2_ in media, followed by incubation at 37 °C for 30 min. The resulting nanoparticles were centrifuged at 13,000 rpm for 10 min. After the supernatant was discarded, the pellet was resuspended in 200 μL mili-Q water. 3 μL of the particle suspension was placed on the glass slide to dry at room temperature before platinum sputtering was applied on the sample. The image was captured through the field-emission SEM (Hitachi S-4700 FE-SEM, Tokyo, Japan).

### 2.5. Generation of Target siRNAs/CA Complexes and Transfection of MCF-7, MDA-MB-231 and 4T1 Cell Lines

Sodium bicarbonate (44 mM) was dissolved with DMEM in an appropriate volume of milliQ water, followed by pH adjustment to 7.4. 3 to 4 μL of 1M CaCl_2_; siRNA at 10 nM, 1 nM, 100 pM, 10 pM and 1 pM was also added to 1 mL of the fresh media. The mixture was then incubated at 37 °C for 30 min. 10% FBS was added to stop further growth of the complexes. Existing culture medium in the 24-well plate was removed and replaced with the prepared medium containing CA-siRNA(s) complexes. Cells were then incubated at 37 °C for 48 h in the incubator humidified with 5% CO_2_.

### 2.6. Cell Viability Assessment with 3-(4,5-Dimethylthiazol-2-yl)-2,5-diphenyltetrazolium Bromide (MTT) Assay

50 μL of MTT solution (5 mg/mL) was added to each well of the 24-well plate following 48 h of cell treatment and incubated for 4 h at 37 °C in the CO_2_ incubator. The media was later removed and 300 μL of DMSO was added. After dissolving crystals and incubating the plate for 5 min at 37 °C, absorbance was measured in a Bio-Rad Benchmark Plus microplate reader (Berkeley, CA, USA) at 595 nm with a reference wavelength of 630 nm. Each experiment was performed in triplicate and data were presented as mean ± standard deviation (SD).

The percentages of cell viability, cytotoxicity and actual cytotoxicity of siRNA(s) were calculated based on the following formulas:

$$\%\;\mathrm{of}\;\mathrm{call}\;\mathrm{viability}=\left(\frac{OD\;treated-OD\;reference}{OD\;untreated-OD\;reference}\right)\times 100$$

% Cytotoxicity=100−% of cell viability

% Actual cytotoxicity (for siRNA)=Cytotoxicity for CA+siRNA−Cytotoxicity for CA

### 2.7. Western Blotting

Treated cells were lysed in 200 µL of the lysis buffer containing 25 mM Tris-HCl (pH 7.4), 150 mM NaCl, 1 mM EDTA, 1% NP-40, 5% glycerol, protease and phosphatase inhibitors (SIGMA). Protein concentrations of cell lysates were determined using Quick Start Bradford Protein Assay kit (Bio-Rad). Equal amount of protein (e.g., 10 µg/lane) was resolved by SDS-PAGE and transferred onto a nitrocellulose membrane via wet transfer technique (Bio-Rad). The membrane was probed with indicated primary antibody (Cell Signaling Technology, Danvers, MA, USA) overnight at 4 °C after blocking with non-fat dry milk in TBST for 1 h at room temperature. Blots were probed with horseradish peroxide-conjugated secondary antibody (Cell Signaling Technology) for 1 h at room temperature, and visualized by using x-ray film (Thermo Scientific, Waltham, MA, USA) and Bio-Rad Gel Document System.

### 2.8. Effects of siRNA-Loaded Nanoparticles on Tumor Regression in 4T1-Induced Breast Cancer Model

Twenty-four female Balb/c mice (6–8 weeks old) of 15–20 g of body weights were purchased and maintained in 12:12 light:dark condition by giving them ad libitum access to food and water. All experiments were done in accordance with the regulations imposed by Monash University Animal Welfare Committee. Approximately 1 × 10^5^ 4T1 cells (in 100 µL PBS) were injected subcutaneously on the mammary pad of mice (considered as day 1) and the mice were checked regularly for outgrowth of tumor by touching the area of injection by index finger. When the volume of the outgrowth tumor reached an average 13.20 ± 2.51 mm^3^, mice were grouped in different assemblies (5 mice per group) randomly and treated intravenously (tail-vein) at the right or left caudal vein. Particles were formed by mixing of 4 μL of 1 M CaCl_2_ in 100 μL of freshly prepared bicarbonated (44 mM) DMEM media and incubating at 37 °C for 30 min. For fabrication of siRNA-loaded particles, 50 nM of a particular siRNA was mixed along with 4 μL of 1 M CaCl_2_ in 100 μL DMEM. The second dose was administered 3 days after the 1st dose. The gross body weights of mice were monitored and the lengths and widths of the outgrowth tumors were measured using the vernier caliper in mm scale for 30 days, while the mice were monitored for their activities. The volume of the tumor was calculated using the following formula:

(1) $$Tumourvolume\left({\mathrm{mm}}^{3}\right)=1/2\left(Length\times Width^{2}\right)$$

The data has been presented here as mean ± SD of tumor volume from each group.

### 2.9. Statistical Analysis

Statistical analysis was done using the SPSS (version 17 for windows) for in vivo (tumor regression study) data. LSD post-hoc test for one way ANOVA was used to analyse and compare the significant difference between different treatment groups. Data was considered statistically significant when * or # *p* < 0.05.

## 3. Results and Discussion

### 3.1. Roles of ER in Proliferation/Survival of Breast Cancer Cells

**Figure 1:**
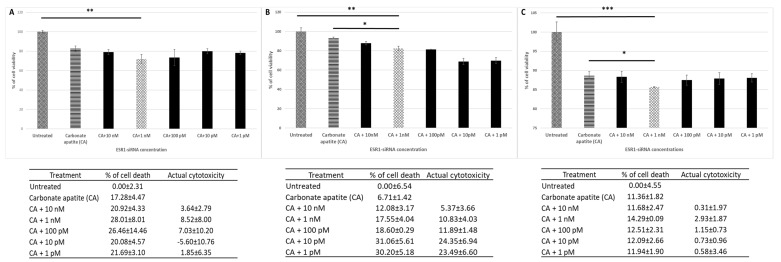
Cell viability and cytotoxicity assessments of MCF-7 cells (**A**), MDA-MB-231 (**B**) and 4T1 (**C**) cells treated with CA-ESR1 siRNA complexes for 48 h. Preparation of CA-ESR1 siRNA complexes involved introduction of different concentrations of ESR1 siRNA (0 to 10 nM) and 3.5 mM of CaCl_2_ to 1 mL of bicarbonate-buffered DMEM (pH 7.4) medium. The mixture was allowed incubation at 37 °C for 30 min prior to addition of 10% FBS. The cells were incubated for the next 48 h with the prepared complexes. MTT assay was performed and absorbance reading was taken at 595 nm with 630 nm as references wavelength. Data was presented as mean ± SD of triplicates. * *p* < 0.05, ** *p* < 0.01 and *** *p* < 0.001.

ER is known to be one of the important biomarkers of breast cancer [7]. In order to investigate its role in proliferation and survival of breast cancer cells, anti-ER (ESR1) siRNA at different concentrations (1 pM–10 nM) was allowed to electrostatically complex with the nanoparticles of CA. Characterization of CA nanoparticles along with assessment of their finding affinity for siRNAs and cellular uptake was provided in our earlier study [18]. The SEM image of the particles has been shown in supplementary Figure S1. Following generation of ESR1 siRNA-loaded CA nanoparticle, the complexes were incubated with MCF-7, MDA-MB-231 and 4T1 cells for a consecutive period of 48 h (Figure 1). The subsequent cell viability assessment showed potent anti-cancer activities of the treatment noticeably in human breast cancer cell lines (MCF-7 and MDA-MB-231) (Figure 1A,B), while in mouse cell line (4T1) the effect was minimal (Figure 1-C), which could be due to higher proliferation rate of the surviving 4T1 cells than those of MCF-7 and MDA-MB-231. Overall 1 nM siRNA concentration seemed to be adequate to bring about a statistically-significant decline in cell viability in all the 3 cell lines tested, apparently by silencing the target mRNA transcript of ER, and therefore used in subsequent experiments. Higher concentration of siRNA might have caused more off-target silencing, while lower concentration of siRNA might not be sufficient to silence the expression of the target gene [18]. ER has thus a significant role in growth/survival of breast cancer cells (Figure 1).

**Figure 2:**
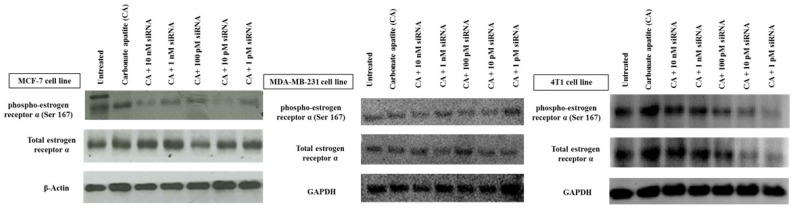
Effects of intracellular delivery of CA-ESR1 siRNA complexes on expression and activation of estrogen receptor α in MCF-7, MDA-MB-231 and 4T1 cell lines. Cells were incubated with CA-ESR1 complexes for 48 h prior to cell lysis for protein extraction and Western blot analysis. Proteins were loaded on SDA-PAGE and transferred onto nitrocellulose membrane for detection of phosphorylated estrogen receptor (ER) α, total ER α and GAPDH (housekeeping protein) expression.

Expression and activation (phosphorylation) of ER following transfection with the target siRNA was found be to down-regulated depending on the siRNA dose, with MCF-7 cells responding most significantly with all siRNA concentrations (Figure 2). Treated MDA-MB-231 cells demonstrated lower effects on reducing the expression and activation of ER at all siRNA doses, while in 4T1 cells clear silencing effects were observed at relatively lower siRNA concentrations (1–100 pM). It should be noted that slight or undetectable change in protein expression does not necessarily indicate that siRNA-mediated knockdown was inefficient, since the protein already expressed before the knockdown could be detectable (depending on its half-life) following siRNA treatment.

### 3.2. Cross-Talks of ER, HER2, EGFR, IGF1R and BCL-2 Signaling Cascades Affecting Proliferation/Survival of Breast Cancer Cells

The engagement of growth factor receptors by their cognate ligands (growth factors) activates multiple signaling cascades that could cross-talk to each other, promoting cancer cells proliferation and survival through activation of MAPK and PI-3 kinase pathways [21]. Both MCF-7 and MDA-MB-231 cells were shown to express ERα, HER2, EGFR and IGF1R [22,23,24,25,26,27], although expression of HER2 was reported to be low in the latter (MDA-MB-231) [28]. In case of 4T1 cells, however, HER2, EGFR and IGF1R were found to be expressed [29,30], while ERα was detected at a low level [31]. Overexpressed in many cancers including breast carcinoma [32], BCL-2 protein, on the other hand, plays an anti-apoptotic role, leading to prolonged cell survival [33,34,35]. Activation of the growth factor receptors, such as HER2 could modulate expression of BCL-2 via activation of PI-3 kinase signaling [36].

**Figure 3:**
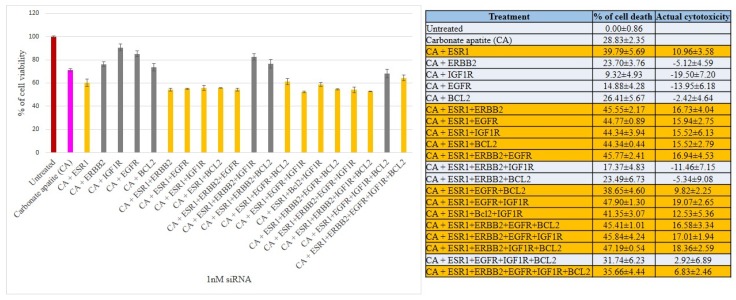
Cell viability and cytotoxicity assessments of MCF-7 cells treated with various CA-siRNA complexes involving ESR1, ERBB2, IGF1R, EGFR and BCL2 siRNAs (1 nM) individually or in combination, for a period of 48 h. Preparation of the CA-siRNA(s) complexes involved introduction of ESR1, ERBB2, IGF1R, EGFR and BCL2 siRNAs individually or in combination with 3.5 mM of CaCl_2_ to 1 mL of bicarbonate-buffered DMEM (pH 7.4). The mixture was allowed incubation at 37 °C for 30 min before the addition of 10% FBS. The cells were incubated for the next 48 h with the prepared complexes. MTT assay was performed and absorbance reading was taken at 595 nm with 630 nm as references wavelength. Data was presented as mean ± SD of triplicates.

**Figure 4:**
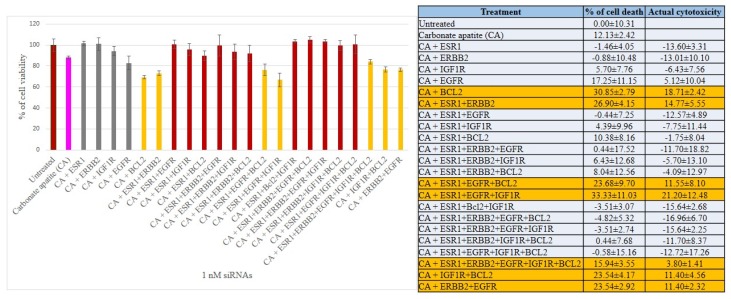
Cell viability and cytotoxicity assessments of MDA-MB-231 cells treated with various CA-siRNA complexes involving ESR1, ERBB2, IGF1R, EGFR and BCL2 siRNAs (1 nM) individually or in combination, for a period of 48 h. Preparation of the CA-siRNA(s) complexes involved introduction of ESR1, ERBB2, IGF1R, EGFR and BCL2 siRNAs individually or in combination with 3.5 mM of CaCl_2_ to 1 mL of bicarbonate-buffered DMEM (pH 7.4). The mixture was allowed incubation at 37 °C for 30 min before the addition of 10% FBS. The cells were incubated for the next 48 h with the prepared complexes. MTT assay was performed and absorbance reading was taken at 595 nm with 630 nm as references wavelength. Data was presented as mean ± SD of triplicates.

**Figure 5:**
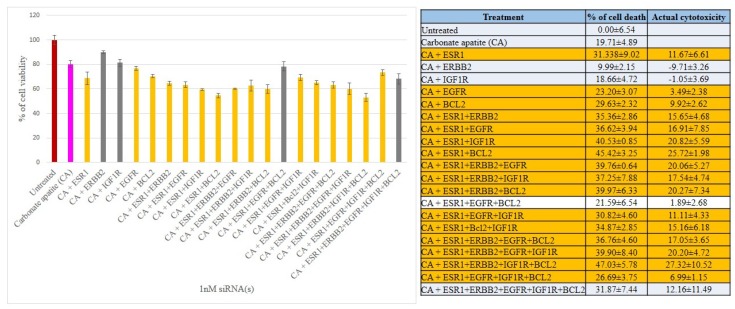
Cell viability and cytotoxicity assessments of 4T1 cells treated with various CA-siRNA complexes involving ESR1, ERBB2, IGF1R, EGFR and BCL2 siRNAs (1 nM) individually or in combination, for a period of 48 h. Preparation of the CA-siRNA(s) complexes involved introduction of ESR1, ERBB2, IGF1R, EGFR and BCL2 siRNAs individually or in combination with 3.5 mM of CaCl_2_ to 1 mL of bicarbonate-buffered DMEM (pH 7.4). The mixture was allowed incubation at 37 °C for 30 min before the addition of 10% FBS. The cells were incubated for the next 48 h with the prepared complexes. MTT assay was performed and absorbance reading was taken at 595 nm with 630 nm as references wavelength. Data was presented as mean ± SD of triplicates.

We have therefore aimed at silencing ER, HER2, EGFR, IGF1R and BCL2 to explore their individual and concerted roles in inducing cell deaths in all three breast cancer cell lines. The CA-siRNA complexes were prepared (as described earlier) with single siRNAs or combinations of the siRNAs (ESR1 siRNA was combined with other siRNAs). As shown in Figure 3, when anti-ESR1 siRNA was delivered in MCF-7 cells along with the siRNAs targeting ERBB2, EGFR, IGF1R or BCL2 (i.e., CA+ESR1+ERBB2, CA+ESR1+EGFR, CA+ESR1+IGF1R or CA+ESR1+BCl2), the cell viability declined to ~85–83% (i.e., ~15–17% cytotoxicity) compared to the individual treatment of CA+ESR1 with ~11% cytotoxicity or CA+ERBB2, CA+IGF1R and CA+BCL2 without any net cytotoxicity, indicating that the signaling pathway of ER cross-talks to that of ERBB2, EGFR or IGFR, or BCL2 molecule. Among the six combinations of 3 siRNAs that were co-delivered, apart from ESRI+ERBB2+IGF1R and ESRI+ERBB2+BCL2 that exerted no net cytotoxicity, other combinations, such as ESRI+ERBB2+EGFR, ESRI+EGFR+BCL2, ESRI+EGFR+IGF1R and ESRI+BCL2+IGF1R exhibited ~10–19% cytotoxicity, suggesting that there was no further significant improvement in cytotoxicity compared to the co-delivery of 2 siRNAs (with one member being ESR1 siRNA), probably owing to the absence of an additional cross-talk among the signaling molecules. Similar results were obtained for treatments of MCF-7 cells with four combinations of 4 siRNAs and one combination of 5 siRNAs, ending up without an enhancement in cytotoxicity (Figure 3). Unlike MCF-7 cells, siRNA targeting BCL2 killed a significant number of MDA-MB-231 cells (representing ~18% cytotoxicity) after CA-facilitated intracellular delivery (Figure 4), indicating that BCL2 is a potential therapeutic target for siRNA-mediated knockdown in this particular breast cancer type. Although CA-ESR1 did not show any cytotoxicity particularly in this independent experiment, it exerted cytotoxic effects in other times, reflecting the variability of effects depending on cell culture condition. Among other combinations involving anti-ESR1 siRNA, CA+ESR1+ERBB2 and CA+ESR1+EGFR+IGF1R led to ~14% and 21% cytotoxicity, respectively, in MDA-MB-231 cells, which is quite similar to the results obtained in MCF-7 cells. On the other hand, 4T1 cells showed a decline in viability following treatment with CA+ESR1, CA+EGFR and CA+BCL2 compared to the nanoparticles alone (Figure 5). A further decrease in viability was observed with actual cytotoxicity ranging from ~15% to ~25% after co-delivery of ESR1 siRNA and the siRNA targeting HER2, IGF1R or BCL2 in 4T1 cells. Co-delivery of 3, 4 or 5 different siRNAs was not accompanied by any more increment in cytotoxicity (Figure 5). In both MCF-7 and 4T1 cells, inclusion of BCL2 siRNA along with the siRNAs against ESR1 and EGFR in CA nanoparticles led to a reduction in cytotoxicity, suggesting an antagonistic role of BCL2 in signaling cascades of ER and EGFR.

### 3.3. MAPK and AKT Expression and Activation Following Treatment with CA-siRNA(s) Complexes

In order to explain the effects of CA+ESR1, CA+ESR1+BCL2, CA+ESR1+IGF1R and CA+ESR1+ERBB2+EGFR in significantly reducing cell viability in reducing viability in both MCF-7 and 4T1 cells (Figure 3 and Figure 5), the expression and activation levels of MAPK and AKT were analyzed by Western blot, following treatments with the nano-formulations of individual and combined siRNAs.

**Figure 6:**
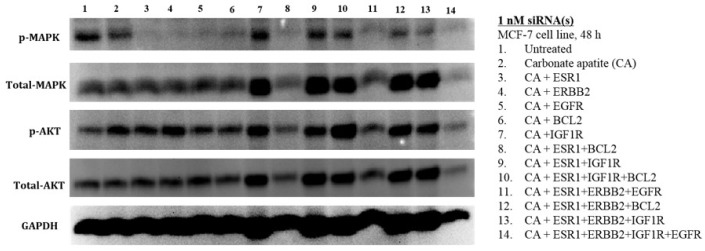
Effects of intracellular delivery of various CA-siRNA complexes on expression and activation of AKT and MAPK proteins in MCF-7 cell line. Preparation of the CA-siRNA(s) complexes involved introduction of ESR1, ERBB2, IGF1R, EGFR and BCL2 siRNAs individually or in combination with 3.5 mM of CaCl_2_ to 1 mL of bicarbonate-buffered DMEM (pH 7.4). The mixture was allowed incubation at 37 °C for 30 min before the addition of 10% FBS. The cells were incubated for a consecutive period of 48 h with the prepared complexes, prior to cell lysis for protein extraction and Western blot analysis. Proteins were loaded on SDS-PAGE and transferred onto nitrocellulose membrane for detection of p-MAPK, p-AKT, total MAPK, total AKT and GAPDH (housekeeping protein) expressions.

Treatment of MCF-7 cells with the single siRNA targeting ER, ERBB2, EGFR, IGF1R or BCL2 was associated with dephosphorylation (deactivation) of MAPK without a change in the levels of total MAPK, total AKT and the phosphorylated form of the latter (Figure 6), thus accounting for the involvement of each of the growth factor receptors and the anti-apoptotic protein (BCL2) in activation of MAPK pathway. However, no enhancement in cytotoxicity upon exposure to CA+ERBB2, CA+EGFR and CA+BCL2 formulations might be owing to the PI-3 kinase pathway, which apparently remained unaffected after the treatments, as evident from the unaltered expression and activation levels of AKT. Among the growth factor receptors, only IGF1R when subjected to knockdown either individually or in combination with other siRNAs, such as ESR1, ESR1+BCL2 or ESR1+ERBB2 led to activation of both AKT and MAPK pathways. In spite of that, when IGF1R siRNA was combined with ESR1 siRNA, the cytotoxicity in MCF-7 cells was found to elevated (Figure 6), suggesting that the cross-talk between the pathways of IGF1R and ER might affect more downstream molecule(s) of MAPK or AKT pathway other than MAPK or AKT, or influence other pathway(s) relating to cell proliferation/viability.

**Figure 7:**
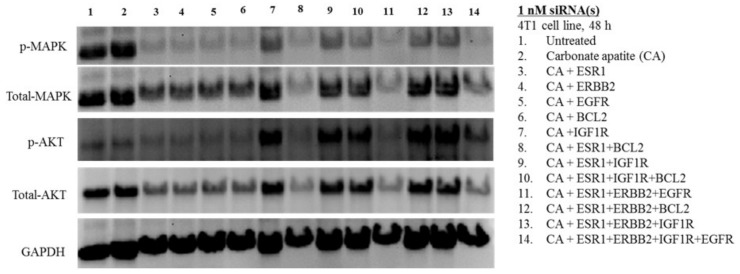
Effects of intracellular delivery of various CA-siRNA complexes on expression and activation of AKT and MAPK proteins in 4T1 cell line. Preparation of the CA-siRNA(s) complexes involved introduction of ESR1, ERBB2, IGF1R, EGFR and BCL2 siRNAs individually or in combination with 3.5 mM of CaCl_2_ to 1 mL of bicarbonate-buffered DMEM (pH 7.4). The mixture was allowed incubation at 37 °C for 30 min before the addition of 10% FBS. The cells were incubated for a consecutive period of 48 h with the prepared complexes, prior to cell lysis for protein extraction and Western blot analysis. Proteins were loaded on SDS-PAGE and transferred onto nitrocellulose membrane for detection of p-MAPK, p-AKT, total MAPK, total AKT and GAPDH (housekeeping protein) expressions.

Almost similar observation in band intensities was made in 4T1 cells, following intracellular delivery of siRNAs using CA nanoparticles (Figure 7). The potent cytotoxic effects of CA+ESR1+BCl2 and CA+ESR1+ERBB2+EGFR in both MCF-7 and 4T1 cells could be explained by their effects on down-regulating both MAPK and AKT pathways (Figure 6 and Figure 7). Similar findings were obtained with CA+ESR1+ERBB2+IGF1R+EGFR. However, compared to MCF-7 cells, the effects of siRNAs (targeting growth factor receptors and BCl2) on suppressing expression and activation of AKT was more evident, which might account for the overall better cytotoxic outcomes in 4T1 cells (Figure 7). In addition, IGF1R siRNA delivery individually or in combination with other siRNAs, resulting in expression and activation of MAPK which was relatively lower in intensity than in MCF-7 cells, which could be responsible for higher toxicity of the nano-formulations containing IGF1R siRNA in 4T1 cells. Furthermore, BCL2 siRNA was shown to exert a stronger effect particularly on downregulating MAPK activation (Figure 7), in proportion to the cytotoxic effects of its various formulations with or without other siRNAs compared to MCF-7 cells (Figure 3 and Figure 5).

**Figure 8:**
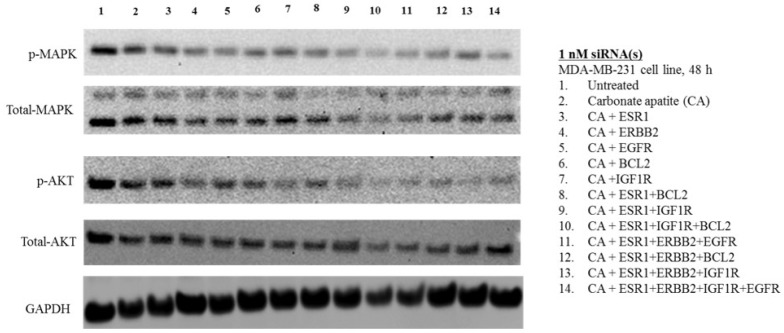
Effects of intracellular delivery of various CA-siRNA complexes on expression and activation of AKT and MAPK proteins in MDA-MB-231 cell line. Preparation of the CA-siRNA(s) complexes involved introduction of ESR1, ERBB2, IGF1R, EGFR and BCL2 siRNAs individually or in combination with 3.5 mM of CaCl_2_ to 1 mL of bicarbonate-buffered DMEM (pH 7.4). The mixture was allowed incubation at 37 °C for 30 min before the addition of 10% FBS. The cells were incubated for a consecutive period of 48 h with the prepared complexes, prior to cell lysis for protein extraction and Western blot analysis. Proteins were loaded on SDS-PAGE and transferred onto nitrocellulose membrane for detection of p-MAPK, p-AKT, total MAPK, total AKT and GAPDH (housekeeping protein) expressions.

Unlike in MCF-7 and 4T1 cells, expression as well as activation of MAPK and AKT was minimally affected (Figure 8) with viability found comparatively lower as a likely consequence, following treatments with different combinations of growth factor siRNAs (Figure 4) in MDA-MB-231 cells. Although BCL2 siRNA delivery resulted in noticeable toxicity in MDA-MB-231 cells, apparently, no change in expression and activation of MAPK and AKT was visible, indicating the involvement of BCL2 as a molecule downstream to MAPK and AKT in the signaling cascades. In contrast to MCF-7 and 4T1 cells, there was upregulation of either MAPK or AKT in terms of both expression and phosphorylation upon exposure to the CA formulations of IGF1R siRNA with or without other siRNAs (Figure 8), justifying the high cytotoxic effect of CA+ESR1+EGFR+IGF1R (Figure 4) in MDA-MB-231 cells.

### 3.4. Intracellular Delivery of Negative Control siRNA Using CA Nanoparticles In Vitro and In Vivo

**Figure 9:**
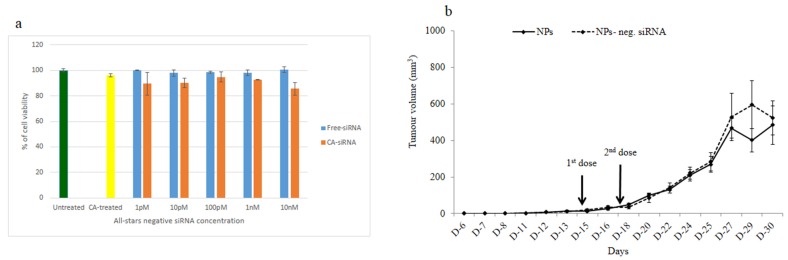
Effect of nanoparticles with loaded negative control siRNA on tumor outgrowth in 4T1-cells induced mouse model. (**a**) After formulation of CA nanoformulation of a negative control siRNA, MCF-7 cells were incubated with it for a consecutive period of 48 h and subsequently, MTT assay was carried out. Mice were treated intravenously through tail-vein injection with 100 µL of either nanoparticles or nanoparticles with electrostatically associated negative control siRNA (50 nM). (**b**) Six mice/group were used and data were represented as mean ± SD.

To rule the possibility of off-target effects of siRNAs in inducing cytotoxicity and tumor regression, we formulated CA nanoformulation of a negative control siRNA that does not target any endogenous mRNA transcript, incubated it for a consecutive period of 48 h with MCF-7 cells and finally, carried out MTT assay in the same way followed for the siRNAs targeting specific cellular genes. As shown in Figure 9a, there was virtually no significant difference in cytotoxicity between the untreated cells and the cell treated with free negative control siRNA, or between the cells treated with nanoparticles and those treated with the nanoparticles loaded with the control siRNA, indicating that enhancement of cytotoxicity by CA nanoparticle complexes of functionally validated siRNAs was due to specific silencing of the target mRNAs in breast cancer cells. Moreover, intravenous delivery of negative control siRNA-loaded nanoparticles twice with a three-day interval into a syngeneic mouse model of 4T1 breast cells did not show any significant difference in tumor outgrowth compared with nanoparticle alone, thus suggesting that tumor regression would not be possible through any off-target effect of a particular siRNA sequence (Figure 9b).

### 3.5. Intravenous Delivery of CA Nanoformulations of siRNAs Targeting ESR1 and BCL2

**Figure 10:**
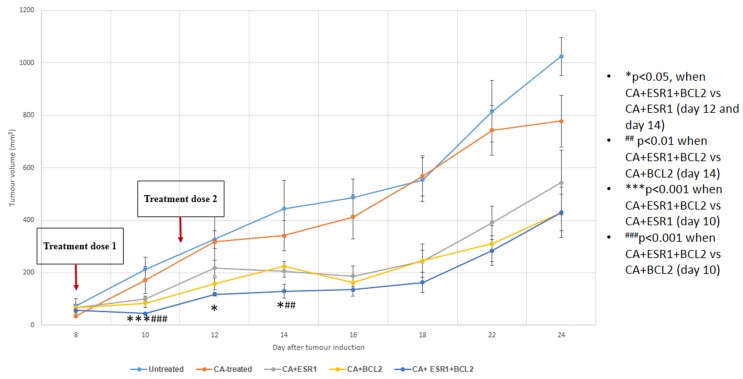
Tumor outgrowth volume of mice intravenously treated with CA+ESR1 siRNA, CA+BCL-2 siRNA and CA+ESR1+BCL-2 siRNA complexes on a 4T1 induced breast tumor mouse model. Mice were administered twice (three days apart) with 100 µL of CA, CA+ESR1 siRNA, CA+BCL-2 siRNA and CA+ESR1+BCL-2 siRNA complexes. CA+siRNA complexes were formed by mixing 50 mM of a particular siRNA along with 4 μL of 1 M CaCl_2_ in 100 μL of freshly prepared bicarbonated (44 mM) DMEM media and incubating at 37 °C for 30 min. Six mice per group were used and data was represented as mean±SD. Values were significant with * *p* < 0.05 compare to the control group.

Based on cytotoxicity study and Western blot analysis, ER and BCL2 have been shown to be potential cellular targets for siRNA-mediated knockdown in effectively reducing proliferation and/or survival of breast cancer cells despite some variations depending on cell types and with regard to single or combined delivery of the siRNAs. To further explore the roles of CA nanoformulations carrying ESR1 and/or BCL2 siRNAs in regressing tumor, mice with 4T1-induced breast tumor was subjected twice to intravenous administrations of the formulations following induction of the tumor. As shown in Figure 10, treatment with CA nanoparticle complex of either anti-ESR1 or anti-BCL2 siRNA significantly reduced the tumor load (*p* < 0.05) in a consecutive manner from day 10 to day 24, confirming the vital role of ER as well as BCL2 in progression (survival and/or proliferation) of 4T1 mammary carcinoma. Moreover, combined delivery of the siRNAs targeting both ER and BCL2 siRNAs showed a trend of further declining the tumor mass, particularly at the earlier stage of the experimental period.

### 3.6. Intravenous Delivery of CA Nanoformulations of siRNAs Targeting ESR1, ERBB2 and EGFR

**Figure 11:**
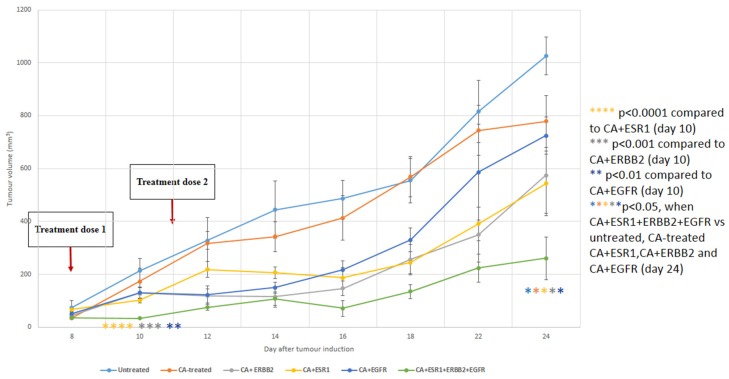
Tumor outgrowth volume of mice intravenously treated with CA+ERBB2 siRNA, CA+ESR1 siRNA, CA+EGFR siRNA and CA+ESR1+ERBB2+EGFR siRNAs complexes on a 4T1 induced breast tumor mouse model. Mice were administered twice (three days apart) with 100 µL of CA, CA+ERBB2 siRNA, CA+ESR1 siRNA, CA+EGFR siRNA and CA+ESR1+ERBB2+EGFR siRNAs complexes. CA+siRNA complexes were formed by mixing 50 mM of a particular siRNA along with 4 μL of 1 M CaCl_2_ in 100 μL of freshly prepared bicarbonated (44 mM) DMEM media and incubating at 37 °C for 30 min. Six mice per group were used and data was represented as mean ± SD. Values were significant with * *p* < 0.05 compare to the control group.

With exception in MDA-MB-231 cells, CA+ESR1+ERBB2+EGFR demonstrated potent cytotoxic effect with suppression of expression and activation of MAPK and PI-3 kinase pathways in MCF-7 cells and more remarkably in 4T1 cells, driving us to examine its outcome in regression of tumor volume following intravenous injection of the formulation in the same syngeneic mouse model of breast carcinoma (Figure 11). Treatment of 4T1 tumors with the single siRNAs targeting either EGFR or HER2 resulted in similar reduction in tumor volume as with ESR1 siRNA, demonstrating the active involvement of these three growth factors in 4T1 tumor growth and/or survival. In addition, combined delivery of the siRNAs against these three growth factor receptors led to a further decline in tumor mass over the entire period except day 14 (Figure 11), suggesting that simultaneous targeting of these three receptors has huge implications for therapeutic intervention in breast cancer.

## 4. Conclusions

In summary, ESR1/BCL-2 and ESR1/ERBB2/EGFR groups of proteins are critically involved in survival and proliferation of MCF-7 and 4T1 breast cancer cells, and CA-based nanoformulations of the siRNAs targeting mRNA transcripts of each of the protein groups led to significant inhibition in tumor growth in a 4T1-induced syngeneic mouse model, thus suggesting their potential implications in therapeutic intervention of breast cancer. The difference in the outcomes from in vitro and in vivo systems could be due to the highly-proliferating cells in tissue culture, which rapidly divide particularly when there is empty space owing to the death of some cells following siRNA delivery. In addition, nanoparticles played a significant role in effective siRNA delivery in the animal model, preventing nuclease-mediated degradation of the siRNAs and eventually, leading to regression of the tumor growth.

## Supplementary Materials

The following are available online at http://www.mdpi.com/2227-9059/6/3/73/s1. Figure S1: Images of CA particles under a scanning electron microscope (SEM), Incorporation of siRNA along with CA caused no further increase in the size (data not shown here). CA nanoparticles were prepared as mentioned in the “materials and methods” section, with the incorporation of appropriate amount of CaCl2 in media, followed by incubation at 37 °C for 30 min. The resulting nanoparticles were centrifuged at 13,000 rpm for 10 min and the supernatant was discarded. The pellet was resuspended in 200 μL mili-Q water. 3 μL of the particle suspension was placed on the glass slide to dry at room temperature. Platinum sputtering was applied on the dried sample and the image was observed through the field-emission SEM. Scale bar, 300 nm.
