# Supplementary material for: siRNAs Targeting Growth Factor Receptor and Anti-Apoptotic Genes Synergistically Kill Breast Cancer Cells through Inhibition of MAPK and PI-3 Kinase Pathways
Source: Biomedicines. 2018 Jun 22;6(3):73. doi: 10.3390/biomedicines6030073 (PMC6164725; doi:10.3390/biomedicines6030073)
Supplement: Supplementary file 1 [file biomedicines-06-00073-s001.pdf]

siRNAs Targeting Growth Factor Receptor and Anti-Apoptotic Genes Synergistically Kill Breast Cancer Cells Through Inhibition of MAPK and PI-3 Kinase Pathways

Nur Izyani Kamaruzman, Snigdha Tiash, Maeirah Ashaie and Ezharul Hoque Chowdhury *

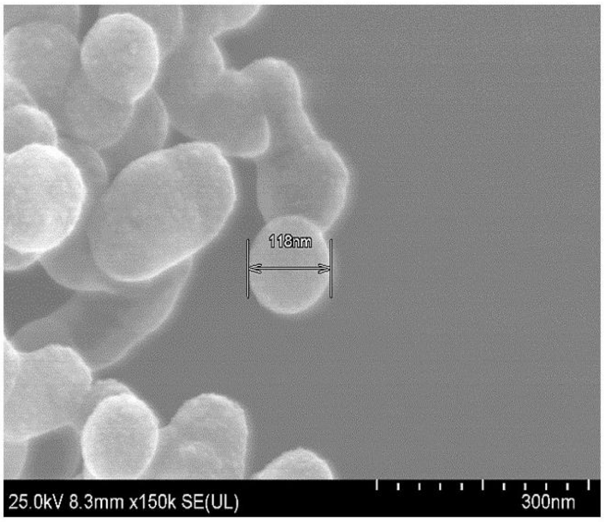

Figure 1: Images of CA particles under a scanning electron microscope (SEM), Incorporation of siRNA along with CA caused no further increase in the size (data not shown here). CA nanoparticles were prepared as mentioned in 'materials and methods' section, with the incorporation of appropriate amount of CaCl2 in media, followed by incubation at 370c for 30 min. The resulting nanoparticles were centrifuged at 13,000 rpm for 10 min and the supernatant was discarded. The pellet was resuspended in 200 μL mili-Q water. 3 μL of the particle suspension was placed on the glass slide to dry at room temperature. Platinum sputtering was applied on the dried sample and the image was observed through the field-emission SEM. Scale bar, 300 nm.
